# Recurrent Necrotizing Enterocolitis in Late Preterm and Full-Term Babies: A Case Report and Literature Review

**DOI:** 10.7759/cureus.80554

**Published:** 2025-03-14

**Authors:** Adel A Alfayez, Zafer Skef

**Affiliations:** 1 Department of Pediatric Surgery, Prince Sultan Military Medical City, Riyadh, SAU; 2 Division of Pediatric Surgery, Department of Surgery, Security Forces Hospital, Riyadh, SAU

**Keywords:** full term neonates, long-term outcome, necrotizing enterocolitis, newborns, recurrent

## Abstract

Necrotizing enterocolitis (NEC) is a serious inflammatory condition of the intestines that primarily affects premature infants, though it can also occur in full-term infants. Diagnosing and managing recurrent NEC in full-term infants can be particularly challenging, with most requiring surgical intervention upon presentation if medical treatment is insufficient. An approach that emphasizes active medical management while minimizing excessive bowel resection tends to result in better postoperative outcomes, highlighting the need for increased awareness within the healthcare community regarding the complexities of NEC cases.

The Security Forces Hospital Program in Riyadh, Saudi Arabia, conducted a study on two cases of necrotizing enterocolitis. The cases involved a full-term baby and a late preterm baby, both of whom had challenging hospital courses and required active care. The study presents a chronological order of the clinical presentation, in-hospital course, and difficulties encountered. The study also includes a brief literature review using PubMed and ProQuest databases.

The first case is of a 34+6-week-old baby boy, weighing 2.26 kg at birth, who experienced a normal vaginal delivery but faced complications due to group B *Streptococcus*. Initially, he showed signs of hypoactivity and a distended abdomen, leading to a diagnosis of sepsis versus necrotizing enterocolitis (NEC). After medical treatment, he was discharged but later returned with worsening symptoms, prompting an ultrasound and subsequent surgery that revealed extensive NEC. Following a stoma procedure and treatment for bowel obstruction, the patient made a full recovery and had a successful stoma closure 10 weeks later. Now, almost four years old, he is thriving and doing well.

In the second case, the mother gave birth to a full-term 39 + 2 weeks-old baby boy via normal spontaneous vaginal delivery, weighing 2.51 kg, with no significant antenatal issues. At 10 days old, the baby developed necrotizing enterocolitis (NEC), which was treated before discharge. However, at two months, he was readmitted with sepsis, and an abdominal X-ray showed a thickened bowel loop but no signs of NEC. An ultrasound later indicated ileocolic intussusception, leading to exploratory surgery that revealed NEC and Meckel's diverticulum, but no intussusception was found. The surgery involved creating stomas, and the baby recovered well, with plans for stoma closure 52 days later. He has since been discharged and is making progress in speech therapy after experiencing some delayed speech.

Recurrent necrotizing enterocolitis is a rare condition in full-term infants, and its occurrence in de novo cases remains unclear. This condition presents significant challenges in diagnosis and management, potentially leading to long-term gastrointestinal issues or even death, highlighting the importance of prompt diagnosis and appropriate treatment. While medical management is the primary approach, surgical intervention may be required in cases where medical treatment fails, and it is crucial to avoid excessive bowel resection and ensure long-term follow-up for affected infants.

## Introduction

The incidence rate of necrotizing enterocolitis (NEC) in full-term neonates is variable. It ranges from one in every 20000 births to 10% of all cases. Prematurity is the most common risk factor for NEC; however, a study conducted in Australia reported an incidence rate of one in 20000 in full-term babies [1].

Eighty-five percent of full-term babies had a cause, like a disease in the mother or the baby itself. Other causes included Hirschsprung's disease and heart problems. Moreover, surgery was more likely to be necessary in full-term neonates with NEC than in the premature population (37.5% versus 22%) [1]. NEC in full-term neonates remains rare, and the incidence rate of NEC in full-term neonates is 10-12.4% [2].

An understanding of the risk factors for NEC in full-term neonates is essential in preventing and managing this pathology. Babies who were born at full term and have necrotizing enterocolitis (NEC) can get acute respiratory distress syndrome (ARDS), sepsis, septic shock, multiple system failure, and low body temperature. Perinatal asphyxia increases the risk of pulmonary hemorrhage, which can cause respiratory deterioration [3]. In addition, antenatal and postnatal conditions cause diminished intestinal blood flow and increase the risk of NEC [4]. 

The development of NEC in full-term neonates typically occurs in the first few days of life, but it can occur anytime in the first two to four weeks [5]. For this reason, infants born with underlying risk factor-related conditions require cautious enteral feeding. Literature rarely documents the recurrence of NEC in late and full-term babies.

Our aim in this paper is to look for the de novo cases and for any commonly known risk factors, estimate the recurrence rate, and review the literature.

## Case presentation

Case 1

This case is of a late preterm 34 + 6 weeks old baby boy with a birth weight of 2.26 kg, delivered via normal spontaneous vaginal delivery. Table 1 shows the detailed presentation of the case. The child’s mother had group B *Streptococcus* (not on treatment).

**Table 1:** Presents the patient characteristics, the chronological sequence of surgical interventions, and their hospital course

	Case 1	Case 2
Gestational age and mode of delivery	34 + 6 weeks, spontaneous vaginal delivery	39 + 2 weeks, spontaneous vaginal delivery
Gender	Male	Male
Birth weight	2.26 Kg	2.51 kg
Risk factors	Maternal: Group B *Streptococcus* positive not treated fetal: none	Maternal: none fetal: none
Age of presentation, and diagnosis	4 days, and 7 weeks, necrotizing enterocolitis	10 days, and 2 months, necrotizing enterocolitis
First surgery	No malrotation or volvulus extensive (NEC) (jejunum, proximal ileum, with areas of frank necrosis). (32 Cm resection + double barrel jejunostomy	No intussusception. Extensive enterocolitis (lower jejunum and proximal ileum). The last 62 cm of the ileum was healthy. Limited small bowel resection 25 cm + de-functioning double barrel ileostomy. Meckle’s diverticulectomy + appendectomy
Second surgery	Matted bowel, severely inflamed, (not separable). Irrigation, culture, and drain insertion.	Closure of stoma
Third surgery	Extensive lysis of adhesions Take down of entero-enteric fistula. Jeujenal stricturoplasty. Sigmoid colon stricture resection and anastomosis. Jeujeno-ileal anastomosis. Appendectomy.	NA
Complications	Bowel obstruction, entero-enteric fistula, and sepsis	None
Preserving versus resectable bowel	Total bowel length (84 Cm). (Included 20 Cm terminal ileum). An intact ileocecal valve.	Total bowel length (150 Cm). (Included 62 Cm terminal ileum). An intact ileocecal valve.
Length of stay (LOS)	First admission: 25 days second admission: 8 months	First admission: 10 days second admission: 60 days
Long term outcome	Oral feeds gaining weight	Oral feeds gaining weight had delayed speech

**Figure 1:**
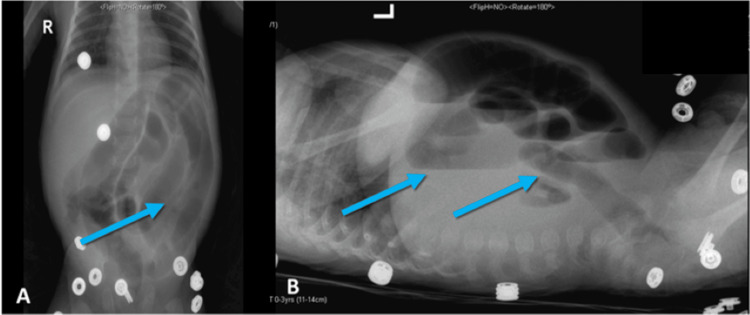
X-ray abdomen, two views: A. The anterior-posterior view showed dilated bowel loops and some pneumatosis intestinalis (blue arrow). B. The lateral decubitus view showed dilated bowel loops and some pneumatosis intestinalis (blue arrows)

**Figure 2:**
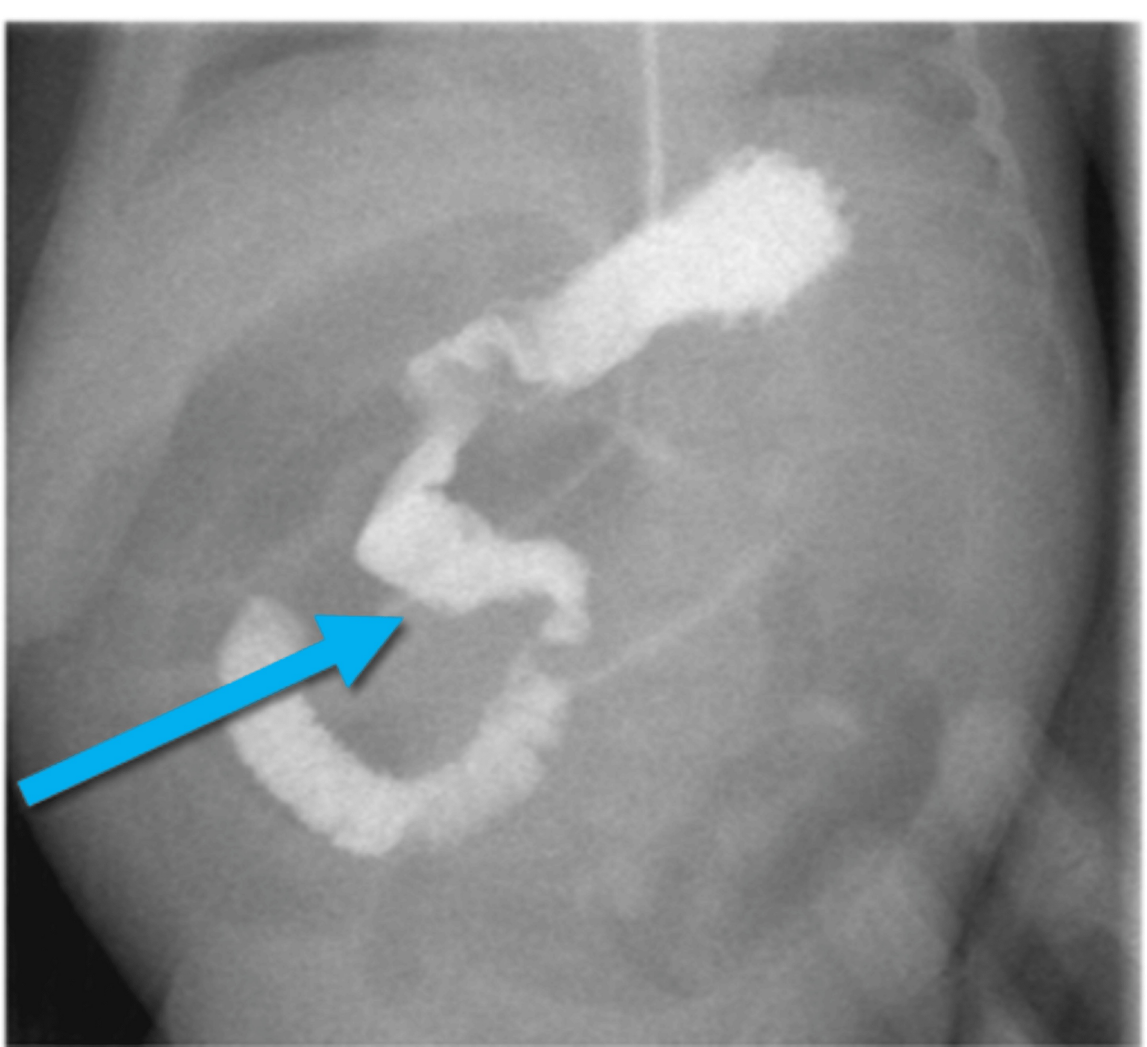
Limited upper gastrointestinal study, showing a picture of intestinal malrotation (incomplete rotation) Duodenojejunal junction not crossing the midline, along with the proximal jejunal loop seen on the sight side (blue arrow).

The child had two attacks of hypoactivity, a distended abdomen, and a picture of sepsis versus NEC, which he was treated for initially, improved with the initiation of feeding gradually, and was discharged home in stable condition. Three weeks later, the patient arrived at our emergency department, presenting with a history of hypoactivity, lethargy, a distended abdomen, and repeated episodes of bilious vomiting. We performed resuscitation, a bedside ultrasound scan (no image), and an upper gastrointestinal contrast study on the patient (Figures 1, 2), and planned for exploration after the imaging.

Imaging revealed findings as described, and the patient was taken for exploration. Table 1 shows the detailed surgical interventions. Later on, the baby recovered well but developed bowel obstruction; for that, he underwent lysis of adhesions and irrigation with drain insertion.

The baby had a successful recovery, and we planned for the final closure of the stoma 10 weeks later. Following the surgery, the patient experienced a smooth recovery, went home in stable condition, thrived well, and underwent follow-up (Table 1).

Case 2

This case is of a full-term 39 + 2-week-old baby boy with a birth weight of 2.50 kg, delivered via normal spontaneous vaginal delivery. Table 1 has a detailed presentation of the case. The mother had an unremarkable antenatal history. At 10 days old, the child developed NEC, for which our hospital managed him outside and discharged him in stable condition. At two months of age, the baby arrived at our hospital with a picture of hypoactivity, poor feeding, fever, a distended abdomen, and sepsis, for which he was treated medically and kept in the intensive care unit.

**Figure 3:**
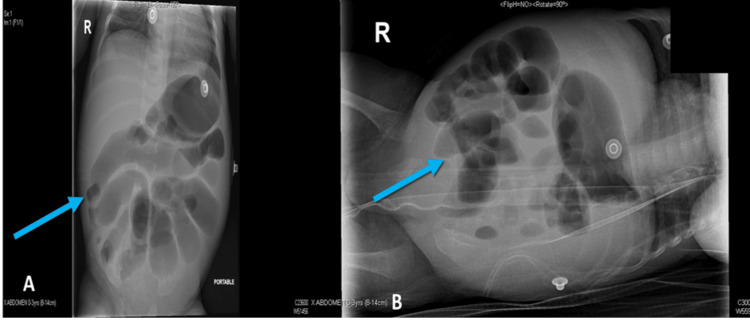
X-ray abdomen, two views: A. Anterior-posterior view showed massive thickened, dilated bowel loops (blue arrow). B. The lateral decubitus view showed no features of pneumatosis intestinalis (blue arrow)

**Figure 4:**
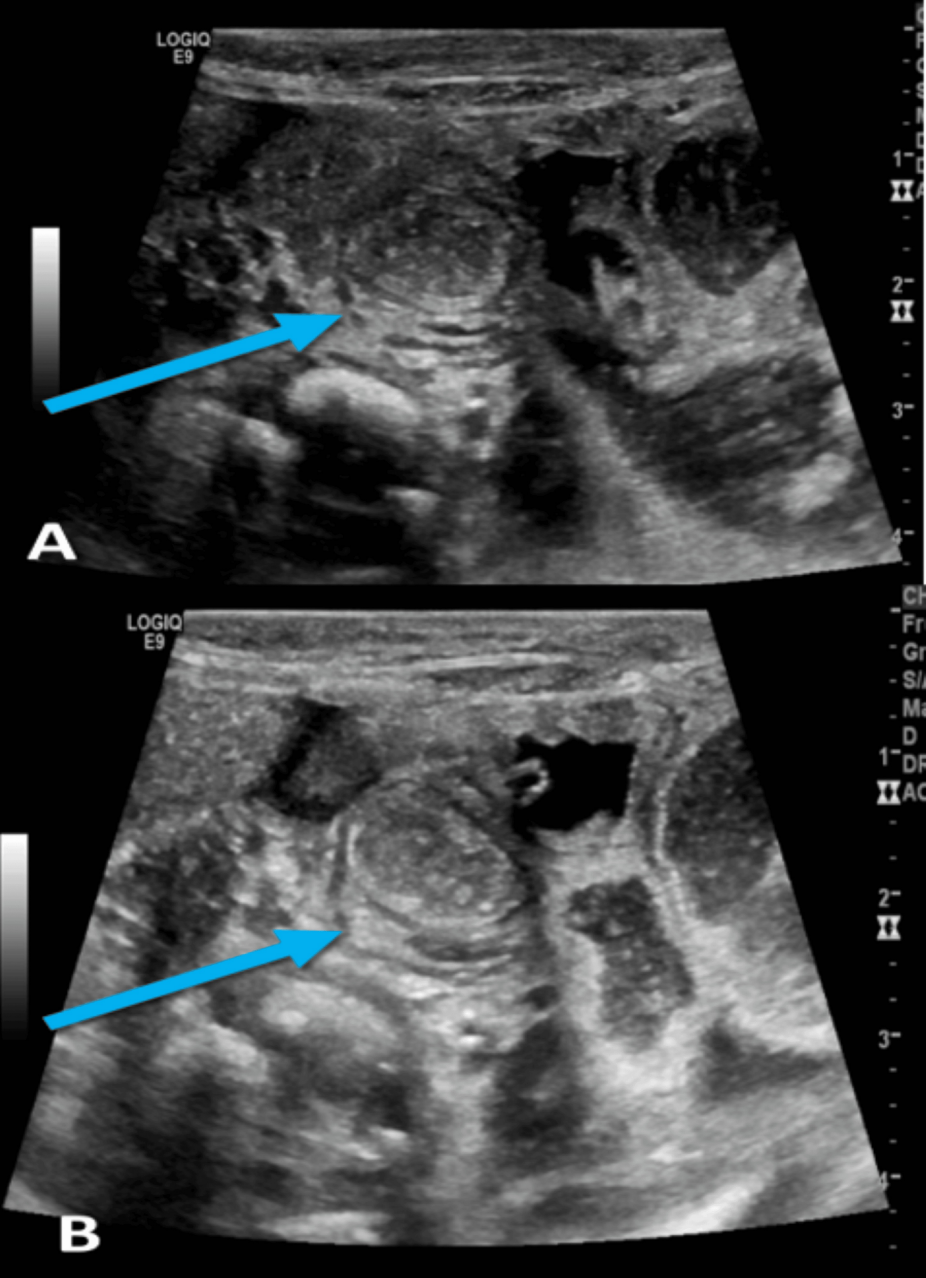
Ultrasound abdomen: A. Cylindrical mass with an outer hypoechoic ring (blue arrow). B. Showing the appearance of a target sign (blue arrow)

X-ray abdomen (Figure 3) revealed a massive thickened dilated bowel loop, no distal gas, and no features of NEC. Moreover, the patient did not improve with medical management, and his condition is getting worse. Ultrasound done at the bedside (Figure 4) revealed a right lower quadrant cylindrical mass with an outer hypoechoic ring surrounding the bowel loop, giving the appearance of a target sign. This finding aligns with the diagnosis of ileocolic intussusception.

On day two post-presentation, we took the patient for exploration (Table 1), which shows the detailed surgical interventions. The baby had a successful recovery and planned for the final closure of the stoma 52 days post-operative.

Postoperatively, the patient recovered well, with uneventful situations, and was discharged home in stable condition, thriving well, and with follow-up (Table 1). At present, the patient has been in good health for three years and is thriving well but has experienced delayed speech, necessitating follow-up with the speech therapist. 

The histopathology report of the specimens is shown in Table 2, the first case (three laparotomies): The first small resection and stoma creation revealed an inflamed and necrotic bowel mucosa, according to pathology. The second pathological specimen from the jejunum, ileum, appendix, and colonic stricture sites was unremarkable, with the presence of ganglion cells. Second case (two laparotomies): Small bowel mucosa, Meckel’s diverticulum, appendix: ulceration and early ischemic changes, ectopic gastric mucosa, ganglion cells present. All are consistent with necrotizing enterocolitis, and Meckel’s diverticulum. Suction rectal biopsy revealed ganglion cells.

**Table 2:** Demonstrate the pathological reports of the presenting cases, in which Hirschsprung disease was ruled out.

Case	Small bowel resection	Stoma site	jejunum	Ileum	Appendix	Colonic stricture site	Rectal biopsy
First(three laparotomies)	Consistent with necrotizing enterocolitis	Unremarkable, with the presence of ganglion cells	Unremarkable, with the presence of ganglion cells	Unremarkable, with the presence of ganglion cells	Unremarkable, with the presence of ganglion cells	Unremarkable, with the presence of ganglion cells	Unremarkable, with the presence of ganglion cells
	Small bowel mucosa	Meckel's diverticulum	Appendix	Suction rectal biopsy			
Second (two laparotomies)	Consistent with necrotizing enterocolitis	Consistent with Meckel’s diverticulum	Unremarkable, with the presence of ganglion cells	Unremarkable, with the presence of ganglion cells			

## Discussion

Necrotizing enterocolitis (NEC) in newborns is a serious condition that requires immediate medical attention. The diagnosis of NEC is based on history and physical examination, symptoms such as bloody stools, abdominal tenderness, increased gastric residuals, abdominal distension, and laboratory tests. Low platelet counts and increased levels of lactate are indicative of NEC. X-rays can also be used to detect intestinal pneumatosis, portal venous gas, pneumoperitoneum, and progression of the disease [6].

Studies have shown that a large proportion of full-term neonates with necrotizing enterocolitis (NEC) have Hirschsprung's disease (HD), which needs to be investigated. We have not previously reported this association between HD and NEC, which is a relatively recent discovery [7].

**Figure 5:**
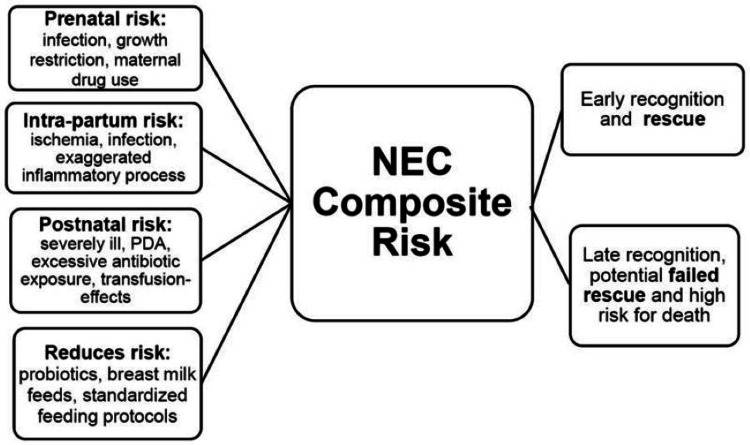
Represent the model of necrotizing enterocolitis risk factors The image is copyright free and is from the following article: Gephardt SM, Effken JA, McGrath JM, Reed PG: Expert consensus building using e-Delphi for necrotizing enterocolitis risk assessment. J Obstetric Gyn. (NEC) Neonatal Nurs. 2013, 42:332-47 Reference citation [4].

In recent years, research has highlighted the frequency of underlying causes in full-term neonates, and these underlying causes include congenital heart disease, Hirschsprung's disease, hypothermia, neonatal hypoxemia, hypoglycemia, and maternal conditions [8] (Figure 5). Hirschsprung's disease-associated enterocolitis (HEC) is a well-known complication of HD and can appear in any age group, especially in the neonatal period [9]. The recurrence rate of NEC in full-term neonates is quite low. Well, it has been found that NEC in full-term babies that is linked to recurrence needs to be looked into, along with finding the underlying risk factor that is usually the cause of recurrence. However, reports of de novo cases, such as those in our study, continue to warrant further investigation.

The primary mode of management for necrotizing enterocolitis (NEC) is medical. Surgery is considered for the management of the complications of the disease (perforation, peritonitis, fistula, obstruction, stricture, etc.). Different procedures are utilized (according to the infant's condition and degree of the disease) such as peritoneal drainage, laparotomy, and resection with or without stoma. Primary laparotomy is the preferred treatment for infants weighing 1000 g or more who have no associated morbidities and are clinically stable. Small, unstable, and critically ill neonates cannot tolerate major surgical intervention, so bedside insertion of a drain is all to be done till the infant's condition improves [10]. 

The objectives of the surgery in near-term or full-term infants are to control sepsis Remove gangrenous bowel and preserve as much bowel length as possible. Avoid aggressive resections to prevent the infant from suffering from short bowel or intestinal failure.

The overall operative rate for NEC increased from 46% to 69% between 1990 and 1999, and this can be attributed to the increase in stage III patients and the greater number of referrals for post-NEC strictures [11]. On the other hand, resection and primary anastomosis are valid treatment options, and up to 50% of neonates with NEC require operative treatment [12].

The long-term effects of NEC can be devastating for both the patient and their family, including death and adverse neurological sequelae. Thus, it is essential to identify and treat NEC early to reduce the potential long-term effects. Conversely, adopting a protocoled feeding regimen and implementing early and active medical versus surgical management can lead to a favorable overall outcome [13].

## Conclusions

Despite its rare occurrence, the literature currently reports multiple cases, each presenting differently. Usually, those patients had an underlying condition, yet cases without an underlying causative factor are present, as in our case. Management includes medical versus surgical interventions, while most of the time those cases need active surgical intervention upon presentation. The postoperative outcome is complex, involving managing the child's nutritional needs, controlling antibiotic regimens, and managing the risks of central line-associated sepsis and liver effects.
